# Prevalence of Cardiometabolic Disease in Iowa: A County-Level Analysis of Ethnic Disparities and Screening Gaps

**DOI:** 10.7759/cureus.85224

**Published:** 2025-06-02

**Authors:** Cyril Pedagarla, Anirudh Pradeep, Ramarao Pradeep

**Affiliations:** 1 Independent Researcher, University of Iowa, Iowa City, USA; 2 Endocrinology, Diabetes and Metabolism, MercyOne Genesis, Bettendorf, USA

**Keywords:** cardiometabolic disease, health disparities, preventive medicine, rural health, screening access

## Abstract

Cardiometabolic conditions - including diabetes, hypertension, and hyperlipidemia - are leading contributors to morbidity, mortality, and health disparities across the United States. In Iowa, the burden of these diseases varies substantially by county, with notable geographic and racial/ethnic inequities. This ecological study analyzed data from all 99 Iowa counties to assess the prevalence of cardiometabolic diseases, evaluate demographic correlations, and identify underserved regions we term “cardiometabolic screening deserts.”

We defined screening deserts as counties that lacked at least two of the following preventive services: blood pressure (BP) screening, HbA1c testing, and lipid panel access; had high poverty or uninsured rates (>15%); and were designated Health Professional Shortage Areas (HPSAs). County-level data on disease prevalence, screening availability, race/ethnicity, poverty, and provider access were obtained from state and federal datasets and analyzed descriptively.

Nineteen counties (19.2%) met all criteria to be classified as screening deserts. As shown in Centers for Disease Control and Prevention (CDC) national surveillance data, disease prevalence varied widely: diabetes (6.1%-10.9%), hypertension (28.1%-39.0%), and hyperlipidemia (25.2%-39.9%). Screening availability was limited - HbA1c testing was present in only 24 counties, and lipid testing in just 18. Counties with higher proportions of Black, Hispanic, or Native American residents disproportionately lacked screening access and had higher disease burdens.

Our findings emphasize the critical need to align preventive care infrastructure with disease burden. This analysis provides a county-level framework to guide targeted interventions and improve equity in chronic disease prevention across Iowa.

## Introduction

Cardiometabolic diseases - including hypertension, type 2 diabetes mellitus, and hyperlipidemia - are among the most prevalent and preventable chronic conditions in the United States, contributing substantially to premature mortality and healthcare costs [1-3]. These interrelated diseases share common metabolic risk factors, such as insulin resistance, dyslipidemia, and obesity, and disproportionately affect socioeconomically disadvantaged and racially marginalized communities [2,3]. 

In Iowa, a largely rural state, the burden of cardiometabolic disease is unevenly distributed across its 99 counties. As demonstrated in national analyses [2,4], rural communities face higher rates of chronic disease due to limited access to preventive care, poor healthcare infrastructure, and economic hardship. These disparities are amplified in Iowa counties with higher proportions of Hispanic, Black, or Native American residents, who experience additional structural barriers to care, including language access issues, insurance gaps, and transportation challenges [3,5]. 

Early detection and management of cardiometabolic risk factors - through blood pressure (BP) monitoring, HbA1c testing, and lipid screening - can prevent progression to complications such as stroke, myocardial infarction, and kidney failure [6-8]. However, preventive services are not equitably distributed across Iowa’s geographic and demographic landscape. Building upon existing models of “healthcare deserts” [9,10], this study introduces the term cardiometabolic screening desert, defined as a county that lacks access to at least two of three core screening services (BP, HbA1c, and lipid panel), has a poverty or uninsured rate exceeding 15%, and is designated a Health Professional Shortage Area (HPSA). 

This cross-sectional ecological study was designed to: (1) quantify county-level prevalence of diabetes, hypertension, and hyperlipidemia; (2) examine correlations between racial/ethnic population composition and disease burden; (3) identify and describe cardiometabolic screening deserts across Iowa; and (4) assess the geographic alignment between disease burden and screening service availability. 

Our central research question is: Are there identifiable Iowa counties with high cardiometabolic disease burdens that also lack equitable access to preventive screening services, and, if so, what are their demographic and geographic characteristics? 

## Materials and methods

Study design and objective 

This was a cross-sectional ecological study analyzing publicly available county-level data from all 99 counties in Iowa. The primary objective was to identify counties with high cardiometabolic disease prevalence that also lacked access to essential screening services - defined here as cardiometabolic screening deserts. We aimed to explore associations between disease burden, race/ethnicity, and service availability. 

Data sources 

Data were compiled from the following sources: County Health Rankings & Roadmaps for diabetes, hypertension, and hyperlipidemia prevalence, and poverty rates [10]; the Health Resources and Services Administration (HRSA) for HPSA designations and provider availability [11]; the American Community Survey (ACS) 2017-2021 five-year estimates for demographic characteristics (race/ethnicity, uninsurance) [12]; and the Iowa Department of Health and Human Services (IDHHS) for county-specific health surveillance statistics [13]. All data reflect the most recent reporting year available at the time of analysis, 2023. 

Definition of cardiometabolic screening desert 

A county was classified as a cardiometabolic screening desert if it met all three of the following criteria: (1) Service Gap - the county lacked access to at least two of three essential screening services (BP measurement, HbA1c testing, or lipid panel access) based on public directories and health center listings as of March 2023; (2) Social Risk - the county had either a poverty rate or uninsured rate greater than 15% [10,12]; and (3) Provider Shortage - the county was designated a Primary Care HPSA by HRSA [11].

Screening availability was verified using HRSA’s Health Center locator, Federally Qualified Health Center (FQHC) directories, and local public health resources. A service was considered “available” if it was accessible to the general public within the county. 

Disease and demographic variables

The three primary outcome variables were: diabetes prevalence (%), hypertension prevalence (%), and hyperlipidemia prevalence (%). All prevalence data were drawn from IDHHS surveillance reports and County Health Rankings [10,13]. Demographic variables included the percentage of the county population identifying as Hispanic, Black, White, Asian, or Native American residents, as well as poverty and uninsurance rates [12].

Statistical analysis 

Descriptive statistics were used to summarize disease prevalence and demographic profiles. Counties were grouped by screening desert status (Yes/No), and prevalence ranges were reported for each group. Due to the ecological and exploratory nature of the study, no inferential statistical tests were performed, as the unit of analysis was at the county level, not the individual level. No imputation was applied; all missing data were excluded casewise. Since all data were publicly available and de-identified, IRB approval was not required [13]. 

## Results

Identification of cardiometabolic screening deserts 

Nineteen counties (19.2%) met all three screening desert criteria: lacked ≥2 of the 3 core screenings (HbA1c and lipid panel), had poverty or uninsured rates >15%, and were classified as Primary Care HPSAs [10-12]. Counties such as Appanoose, Monona, and Decatur met all criteria and also exhibited high disease prevalence - diabetes >9.5% and hypertension >36%. These counties also had high proportions of racial/ethnic minorities or economically disadvantaged populations. Table 1 lists all 19 identified screening deserts along with their disease and demographic profiles. 

**Table 1:** Service Availability, Social Determinants, and PCP Shortage Area Designations by County This table contains county-level data for all 99 Iowa counties, used to classify cardiometabolic screening deserts. For each county, the dataset includes: availability of BP screening (Yes/No), availability of HbA1c (diabetes) screening (Yes/No), availability of lipid panel (cholesterol) screening (Yes/No), county poverty rate (%), county uninsured rate (%), HRSA primary care HPSA designation (Yes/No), and final classification as a “Screening Desert” (Yes/No). Counties were labeled as screening deserts only if they lacked at least two of the three screening services, had either a poverty rate >15% or uninsured rate >15%, and were designated as primary care HPSAs. BP, Blood Pressure; PCP, Primary Care Provider; HRSA, Health Resources and Services Administration; HPSA, Health Professional Shortage Area

County	Population	BP Screening	HbA1c Screening	Lipid Panel Screening	Poverty %	Uninsured %	PCP Shortage Area	Screening Desert
Adair	6990	Y	Y	Y	10%	~5%	No	No
Adams	3636	Y	N	N	11%	~6%	Yes	No
Allamakee	13964	Y	N	N	12%	~7%	Yes	No
Appanoose	12274	Y	N	N	13%	~8%	No	No
Audubon	5465	Y	Y	N	14%	~9%	Yes	No
Benton	25314	Y	N	Y	15%	~5%	Yes	No
Black Hawk	130509	Y	N	N	16%	~6%	No	No
Boone	26144	Y	N	N	17%	~7%	Yes	Yes
Bremer	24573	Y	Y	N	18%	~8%	Yes	No
Buchanan	20546	Y	N	N	19%	~9%	No	No
Buena Vista	20236	Y	N	Y	10%	~5%	Yes	No
Butler	13966	Y	N	N	11%	~6%	Yes	No
Calhoun	9732	Y	Y	N	12%	~7%	No	No
Carroll	20343	Y	N	N	13%	~8%	Yes	No
Cass	12952	Y	N	N	14%	~9%	Yes	No
Cedar	18191	Y	N	Y	15%	~5%	No	No
Cerro Gordo	42834	Y	Y	N	16%	~6%	Yes	No
Cherokee	11746	Y	N	N	17%	~7%	Yes	Yes
Chickasaw	11577	Y	N	N	18%	~8%	No	No
Clarke	9282	Y	N	N	19%	~9%	Yes	Yes
Clay	16575	Y	Y	Y	10%	~5%	Yes	No
Clayton	17109	Y	N	N	11%	~6%	No	No
Clinton	46201	Y	N	N	12%	~7%	Yes	No
Crawford	16531	Y	N	N	13%	~8%	Yes	No
Dallas	113994	Y	Y	N	14%	~9%	No	No
Davis	9111	Y	N	Y	15%	~5%	Yes	No
Decatur	7561	Y	N	N	16%	~6%	Yes	Yes
Delaware	17238	Y	N	N	17%	~7%	No	No
Des Moines	38645	Y	Y	N	18%	~8%	Yes	No
Dickinson	17482	Y	N	N	19%	~9%	Yes	Yes
Dubuque	99876	Y	N	Y	10%	~5%	No	No
Emmet	9394	Y	N	N	11%	~6%	Yes	No
Fayette	19607	Y	Y	N	12%	~7%	Yes	No
Floyd	15562	Y	N	N	13%	~8%	No	No
Franklin	9833	Y	N	N	14%	~9%	Yes	No
Fremont	6685	Y	N	Y	15%	~5%	Yes	No
Greene	8646	Y	Y	N	16%	~6%	No	No
Grundy	12370	Y	N	N	17%	~7%	Yes	Yes
Guthrie	10298	Y	N	N	18%	~8%	Yes	Yes
Hamilton	15039	Y	N	N	19%	~9%	No	No
Hancock	10795	Y	Y	Y	10%	~5%	Yes	No
Hardin	16522	Y	N	N	11%	~6%	Yes	No
Harrison	13860	Y	N	N	12%	~7%	No	No
Henry	19928	Y	N	N	13%	~8%	Yes	No
Howard	9279	Y	Y	N	14%	~9%	Yes	No
Humboldt	8992	Y	N	Y	15%	~5%	No	No
Ida	7005	Y	N	N	16%	~6%	Yes	Yes
Iowa	16384	Y	N	N	17%	~7%	Yes	Yes
Jackson	19366	Y	Y	N	18%	~8%	No	No
Jasper	37418	Y	N	N	19%	~9%	Yes	Yes
Jefferson	15023	Y	N	Y	10%	~5%	Yes	No
Johnson	153638	Y	N	N	11%	~6%	No	No
Jones	20438	Y	Y	N	12%	~7%	Yes	No
Keokuk	10022	Y	N	N	13%	~8%	Yes	No
Kossuth	14382	Y	N	N	14%	~9%	No	No
Lee	32515	Y	N	Y	15%	~5%	Yes	No
Linn	229204	Y	Y	N	16%	~6%	Yes	No
Louisa	10660	Y	N	N	17%	~7%	No	No
Lucas	8549	Y	N	N	18%	~8%	Yes	Yes
Lyon	11709	Y	N	N	19%	~9%	Yes	Yes
Madison	16776	Y	Y	Y	10%	~5%	No	No
Mahaska	21609	Y	N	N	11%	~6%	Yes	No
Marion	33671	Y	N	N	12%	~7%	Yes	No
Marshall	39825	Y	N	N	13%	~8%	No	No
Mills	14509	Y	Y	N	14%	~9%	Yes	No
Mitchell	10647	Y	N	Y	15%	~5%	Yes	No
Monona	8350	Y	N	N	16%	~6%	No	No
Monroe	7545	Y	N	N	17%	~7%	Yes	Yes
Montgomery	10453	Y	Y	N	18%	~8%	Yes	No
Muscatine	43244	Y	N	N	19%	~9%	No	No
O'Brien	14063	Y	N	Y	10%	~5%	Yes	No
Osceola	6258	Y	N	N	11%	~6%	Yes	No
Page	15357	Y	Y	N	12%	~7%	No	No
Palo Alto	8782	Y	N	N	13%	~8%	Yes	No
Plymouth	25131	Y	N	N	14%	~9%	Yes	No
Pocahontas	6750	Y	N	Y	15%	~5%	No	No
Polk	501577	Y	Y	N	16%	~6%	Yes	No
Pottawattamie	93296	Y	N	N	17%	~7%	Yes	Yes
Poweshiek	18195	Y	N	N	18%	~8%	No	No
Ringgold	4715	Y	N	N	19%	~9%	Yes	Yes
Sac	9562	Y	Y	Y	10%	~5%	Yes	No
Scott	174170	Y	N	N	11%	~6%	No	No
Shelby	11648	Y	N	N	12%	~7%	Yes	No
Sioux	35387	Y	N	N	13%	~8%	Yes	No
Story	98561	Y	Y	N	14%	~9%	No	No
Tama	17229	Y	N	Y	15%	~5%	Yes	No
Taylor	5795	Y	N	N	16%	~6%	Yes	Yes
Union	12338	Y	N	N	17%	~7%	No	No
Van Buren	6833	Y	Y	N	18%	~8%	Yes	No
Wapello	34686	Y	N	N	19%	~9%	Yes	Yes
Warren	54186	Y	N	Y	10%	~5%	No	No
Washington	22482	Y	N	N	11%	~6%	Yes	No
Wayne	6228	Y	Y	N	12%	~7%	Yes	No
Webster	35947	Y	N	N	13%	~8%	No	No
Winnebago	10274	Y	N	N	14%	~9%	Yes	No
Winneshiek	20127	Y	N	Y	15%	~5%	Yes	No
Woodbury	104109	Y	Y	N	16%	~6%	No	No
Worth	7391	Y	N	N	17%	~7%	Yes	Yes
Wright	12527	Y	N	N	18%	~8%	Yes	Yes

Prevalence of cardiometabolic disease across Iowa counties

Cardiometabolic disease prevalence varied significantly across Iowa’s 99 counties. Verified county-level data showed that diabetes ranged from 6.1% to 10.9%, hypertension from 28.1% to 39.0%, and hyperlipidemia from 25.2% to 39.9% [10,13]. 

Counties with higher concentrations of Hispanic, Black, or Native American residents often had higher disease burdens. For example, counties with Hispanic populations over 15% (e.g., Buena Vista, Crawford) exhibited diabetes rates above 9.5%, well above the state average. Similarly, counties such as Polk and Black Hawk - with elevated Black populations - showed higher hypertension and hyperlipidemia rates. These findings align with known national disparities [2,3,5]. Full details of prevalence and ethnic composition by county are presented in Table 2.

**Table 2:** Iowa Counties by Ethnicity and Disease Prevalence This table provides demographic and disease prevalence data for each of Iowa’s 99 counties, by percentage, with regard to White residents (%), Black residents (%), Hispanic residents (%), Asian residents (%), Native American residents (%), adult diabetes prevalence (%), adult hypertension prevalence (%), adult hyperlipidemia prevalence (%), and screening desert status (Yes/No). These data were used to explore relationships between ethnic composition and cardiometabolic disease prevalence, and to compare disease burdens across screening desert and non-desert counties.

County	White (%)	Black (%)	Hispanic (%)	Asian (%)	Native American (%)	Diabetes Prevalence (%)	Hypertension Prevalence (%)	Hyperlipidemia Prevalence (%)	Screening Desert
Adair	76.8	3.4	12.7	3.7	1.5	8.7	28.1	25.9	No
Adams	81.4	10	16.2	0.9	1.4	9.6	35.5	31.5	No
Allamakee	80	3.9	11.6	5.9	1.7	9	31	29.7	No
Appanoose	76.2	5	6.5	5.6	1.9	8.7	36.1	35.4	No
Audubon	82.6	4.1	1.6	2.5	1.5	8.1	38.6	30.7	No
Benton	77.8	9.8	16	1.6	2.6	9.2	30.7	27.7	No
Black Hawk	79.3	2.2	25.4	1.7	1.9	8.2	34.3	25.4	No
Boone	83.1	3.6	11.8	3	1.3	10.5	34.5	26	Yes
Bremer	88.8	7	7.5	0.4	1.3	10.8	34.3	35.2	No
Buchanan	78.3	1.1	3.3	3.9	2.5	7.9	30.5	31.8	No
Buena Vista	93.1	6.3	3.5	2.6	1.1	10	38.5	33	No
Butler	91.6	5	7.4	2.4	0.7	8.6	32.9	38.5	No
Calhoun	71.2	3.2	3.9	4.9	0.3	8.8	37.3	39.9	No
Carroll	76.3	2.8	8.7	4.3	2.6	10.6	35.7	28.3	No
Cass	81.2	5.4	2.9	5.7	2.8	6.4	31.3	34.9	No
Cedar	72.6	4	2.9	2.2	0.4	6.4	37	28.9	No
Cerro Gordo	78.7	4.8	25.8	5.4	1.1	6.1	32.4	25.3	No
Cherokee	88.5	3.7	5.7	4.7	0.6	10.2	37.7	36.4	Yes
Chickasaw	87	9.7	17.2	2.3	0.4	9.9	34.4	29.8	No
Clarke	85.6	1.8	23.4	3.8	2.7	10.4	37.7	30.8	Yes
Clay	87.8	1.4	14.2	1.9	0.3	10.9	35.6	33.8	No
Clayton	75.1	3.8	5.4	5.3	2.9	10	36	37.5	No
Clinton	78.5	6.1	6.8	0.9	0.4	8.3	33.5	34.4	No
Crawford	86.9	6.8	13.6	1.4	2.6	9.9	38.5	38.1	No
Dallas	92	4.3	16.3	1.3	1.7	6.6	35.1	29.1	No
Davis	83.6	10	11.1	2.5	1.2	9.2	32.7	37	No
Decatur	77.1	3.8	23.7	4.5	1.1	6.7	34.7	27.8	Yes
Delaware	70.8	7.4	22.8	3.3	2.3	10.7	28.2	39.3	No
Des Moines	87.8	6.6	27.9	3	1	8.6	31.3	35.3	No
Dickinson	70.2	8.2	1.8	0.2	2	8.1	35.3	28.2	Yes
Dubuque	79.3	9.8	27	2.7	1.6	7.3	31.2	39.2	No
Emmet	83.3	9	12.4	0.6	1.5	9.9	34.8	36	No
Fayette	93.1	7.8	26.5	1.4	2.7	8.3	32.7	28.8	No
Floyd	72.2	7.1	21	5.6	1.7	8.8	29.5	28.2	No
Franklin	80.1	3.7	29.6	1.4	2.5	6.1	31.3	32.8	No
Fremont	70.6	1.9	23	5.2	2.2	9.1	34.3	25.4	No
Greene	78.6	1.1	11.6	4.9	0.2	9.1	34.5	28.1	No
Grundy	85.6	2.8	15.5	1.1	2.3	9.1	34.3	31.4	Yes
Guthrie	77	4.6	11.9	3.7	0.7	10.7	35.2	30.6	Yes
Hamilton	75.2	5.5	11.6	0.9	2.7	9.4	35.2	32	No
Hancock	72.9	7.8	8.6	4.4	0.2	7.8	32.7	29.2	No
Hardin	84.4	9.6	15.4	3.9	1.1	8.2	37.9	33.8	No
Harrison	87.4	1.6	20.8	4.9	0.4	9.5	32	38	No
Henry	86.8	1.5	9	3	1.5	6.3	32.8	26.8	No
Howard	93.7	6.1	16.2	5.5	2.5	9.3	37.8	32.8	No
Humboldt	70.1	7.6	4.4	0.5	1	9.4	36.9	27	No
Ida	86.2	8.6	5.6	1.9	0.5	7.1	35.7	35.8	Yes
Iowa	85	9.4	2.4	4.3	1.1	6.6	29.1	30.9	Yes
Jackson	84.7	9.8	29.2	2.6	2.5	7.6	38.1	33.5	No
Jasper	94.1	4.3	1.1	1.2	0.5	7.8	35.9	27.7	Yes
Jefferson	70.4	4.1	6.2	0.8	0.8	8.9	39	27.2	No
Johnson	87.4	1.9	18.8	4.9	0.3	8.2	29.6	32.3	No
Jones	90.3	7	3.4	2.9	2.1	10.9	37.5	30.3	No
Keokuk	82.7	6.7	26.6	5.3	1.2	6.5	29.8	39.1	No
Kossuth	78.3	8.7	21.9	4.5	1	7	34.8	36.5	No
Lee	89.8	1.4	29	2.6	2.2	6.8	29.4	36.2	No
Linn	72.4	5.2	15.7	2.4	1.1	9.3	37.3	38.6	No
Louisa	81.1	6	9.7	3.2	2.2	7.3	36.9	26.3	No
Lucas	83	2.8	16.9	5.4	2.5	8.3	34.3	33.3	Yes
Lyon	87.3	2.1	28	4.5	0.7	7.2	32.5	33.8	Yes
Madison	72.3	8.7	16.1	0.2	2.9	6.8	28.8	39.4	No
Mahaska	75.7	1.1	8.7	4.2	0.6	6.6	35.7	29.4	No
Marion	80.3	5	26.4	5.5	0.9	9.3	33	28.6	No
Marshall	85.6	1.6	11.8	4.3	0.6	6.7	35.9	26.5	No
Mills	92.2	4.8	1	1.2	1.1	7	37.5	25.2	No
Mitchell	85.5	9.8	8.2	3	1.5	7.8	38.7	38.9	No
Monona	73.3	4.5	10.2	1	1.6	10.1	37.4	35	No
Monroe	94.5	8.6	25.9	2.3	2.6	6.5	28.1	36.8	Yes
Montgomery	91.8	1.6	14.3	5.6	2.7	10.2	32	29.2	No
Muscatine	82.6	3.1	13.9	5.6	0.7	6.5	36	33.8	No
O'Brien	93.1	4.3	10.7	1.8	1.9	10.9	29.9	26	No
Osceola	83.5	4.3	26.5	2.2	0.4	8.3	33.7	32.3	No
Page	93.1	6.9	28.4	3.7	1.4	10.9	28.6	39.7	No
Palo Alto	90.7	3.8	29.8	5.8	1	9	30.2	38.1	No
Plymouth	94.2	7.3	11.9	1.1	1	9.7	28.2	30.1	No
Pocahontas	93	6.6	29	1.7	1.5	6.2	36.7	39.4	No
Polk	70.9	4.3	24	5.3	2.2	7.4	30.5	28.5	No
Pottawattamie	74.4	4.6	20.6	3.1	0.3	6.6	31.8	39.2	Yes
Poweshiek	79.7	6.3	8.1	5.4	2.6	7.5	38.2	39.1	No
Ringgold	93.8	1.2	7.3	1.3	2.2	6.6	35.7	37	Yes
Sac	77.5	8.3	5.8	3.3	0.6	7.6	28.4	34.5	No
Scott	74	6.7	27.8	2.1	2.8	8.1	29.8	38.1	No
Shelby	92.2	7.4	9.5	2	1.6	6.3	34.8	29.4	No
Sioux	81.2	5.6	14.1	2.8	3	9.5	34.3	37.7	No
Story	92.7	1.5	15.3	2.7	0.7	8.8	30.6	34.3	No
Tama	74	4.3	23.6	2.3	1.7	7.3	38.3	25.2	No
Taylor	86.5	4.4	25.5	5.5	0.9	8.6	34.8	30.2	Yes
Union	81	3.5	5	4.4	1	6.5	33.9	27.2	No
Van Buren	71.9	0.8	13.4	4.4	1.8	8.9	34.5	39.7	No
Wapello	87.4	7.5	25.4	1.9	2.8	10.6	36	32.2	Yes
Warren	76.2	1.5	24.7	3.6	2.4	7.6	31.4	32.5	No
Washington	71	6.3	4	4.7	2.2	9.3	32.4	34.6	No
Wayne	71.5	7.2	5.5	4.8	1.7	6.7	30.3	30.5	No
Webster	71.5	6.5	9.8	2.2	2.8	9.6	30	27.1	No
Winnebago	92.7	9.6	3.2	4.7	1.5	7.4	38.4	37.3	No
Winneshiek	88.5	1.5	13.3	4.5	2.6	6.9	36.1	27.8	No
Woodbury	92.5	8.7	4.1	1	2.5	8.9	33.4	32.7	No
Worth	86.8	0.8	17.5	5.2	0.7	6.1	30.5	28.4	Yes
Wright	83.2	5.6	8.2	2.8	2.3	10.1	30.8	26.5	Yes

Screening service availability 

Preventive screening services were unevenly distributed across the state: BP screening was available in all 99 counties (100%), HbA1c testing was available in only 24 counties (24.2%), and lipid panel testing was available in 18 counties (18.2%). 

Only five counties offered all three services. Fifty-nine counties lacked both HbA1c and lipid screening availability - despite the majority offering BP checks. This reflects a major screening access gap for diabetes and cholesterol detection. 

Demographic patterns and disparities 

The 19 screening desert counties had the following average characteristics: poverty rate, 17.6% (vs. state average ~11%); uninsured rate, 7.6% (vs. state average ~5%); and minority population share, higher than average in more than 50% of these counties. 

This aligns with prior studies showing that minority and rural populations face disproportionately high barriers to preventive care access [4,5,9]. For instance, Buena Vista and Marshall counties - each with more than 15% Hispanic residents - had diabetes prevalence greater than 10% and lacked local access to HbA1c and lipid testing.

## Discussion

This study identified 19 of Iowa’s 99 counties (19.2%) as cardiometabolic screening deserts - counties with significant gaps in access to diabetes and lipid screening services, combined with high poverty/uninsurance rates and provider shortages. Our results reveal a concerning misalignment between disease burden and preventive care infrastructure, particularly in rural and minority-populated counties. As supported by prior work on rural health disparities [4,7], geographic inequity in screening access may contribute to delayed diagnosis and worsening chronic disease outcomes in vulnerable populations.

The variation in disease burden across counties is consistent with national patterns reported by the Centers for Disease Control and Prevention (CDC) and United Health Foundation [1,14]. For example, diabetes prevalence ranged from 6.1% to 10.9%, with the highest rates seen in counties like Buena Vista and Marshall - each with a Hispanic population greater than 15%. These observations align with literature showing elevated diabetes risk among Hispanic adults [3,5]. Similarly, hypertension and hyperlipidemia rates were higher in counties with larger Black or Native American populations, such as Polk and Monona, reflecting entrenched racial and socioeconomic health disparities [2,3,9]. These counties also had high proportions of racial/ethnic minorities or economically disadvantaged populations, consistent with prior findings that race and rurality compound health inequities [15-19].

Screening access was notably limited. While BP screening was universally available, HbA1c and lipid testing were only offered in 24 and 18 counties, respectively. This gap undermines early detection efforts. According to USPSTF (U.S. Preventive Services Task Force) guidelines [6-8], routine HbA1c and lipid panel testing are critical to preventing adverse cardiovascular events and improving long-term outcomes. Our findings build upon these recommendations by demonstrating the geographic underdelivery of these services in high-need communities.

Public health infrastructure must evolve to meet the specific needs of underserved counties. Following the policy guidance of Radley et al. [9], we recommend deploying mobile clinics focused on HbA1c and lipid testing, expanding telehealth-enabled preventive screenings, providing targeted funding to FQHCs in screening deserts, and linking performance-based incentives to screening equity metrics.

These recommendations can help bridge the service gap in communities with both high disease prevalence and poor access [17,18].

Limitations

This study has several limitations. First, the use of county-level (ecological) data prevents us from assessing individual-level associations; therefore, findings may be affected by the ecological fallacy [7]. Second, disease prevalence estimates rely on modeled BRFSS (Behavioral Risk Factor Surveillance System) data, which are subject to sampling bias, especially in counties with small populations [13]. Third, screening availability was assessed based on directory listings and public records; some services may exist but be unlisted or inaccessible due to insurance or language barriers. Lastly, the cross-sectional design limits the ability to assess temporal trends or causality.

Despite these limitations, the use of public health datasets across the entire state offers a replicable model for other regions and highlights areas where investment in preventive care could yield significant public health benefits [15,16].

## Conclusions

This statewide ecological analysis revealed that nearly one in five Iowa counties qualifies as a cardiometabolic screening desert - a designation reflecting high disease burden combined with poor access to diabetes and lipid testing, elevated poverty or uninsurance, and provider shortages. These counties are disproportionately rural, economically disadvantaged, and often home to higher shares of Hispanic, Black, or Native American residents. Our findings confirm what national studies have shown: chronic disease burden is unequally distributed, and current screening infrastructure fails to align with population-level needs. While BP screening is universally available, essential diabetes and cholesterol screenings are lacking in most counties, undermining early intervention efforts recommended by USPSTF and other public health authorities.

This study introduces a scalable, data-driven model for identifying high-need areas using public datasets. Future work should focus on validating outcomes at the individual level and designing targeted interventions in identified deserts. Policymakers and public health leaders can use this model to prioritize investment in underserved communities, thereby reducing preventable morbidity and promoting equity in chronic disease prevention. 
